# Psychosocial Impact of Malocclusion and Self-Perceived Orthodontic Treatment Need among Young Adult Dental Patients

**DOI:** 10.1055/s-0042-1753452

**Published:** 2022-09-08

**Authors:** Afnan A. Ben Gassem, Aljazi H. Aldweesh, Eman I. Alsagob, Aljawharah M. Alanazi, Arwa M. Hafiz, Rahaf S. Aljohani, Yara E. Kurdi, Osama Abu Hammad

**Affiliations:** 1 Department of Pediatric Dentistry and Orthodontics, College of Dentistry, Taibah University, Al-Madinah Al-Munawwarah, Saudi Arabia; 2 Department of Pediatric Dentistry and Orthodontics, Dental College, King Saud University, Riyadh, Saudi Arabia; 3 Preventive Dental Sciences Department, College of Dentistry, Princess Nourah Bint Abdulrahman University, Riyadh, Saudi Arabia; 4 College of Dentistry, Taibah University, Al-Madinah Al-Munawarah, Saudi Arabia; 5 School of Dentistry, University of Jordan and Taibah University, Al-Madinah Al-Munawwarah, Saudi Arabia

**Keywords:** malocclusion, perceptions, oral health-related quality of life, Dental Aesthetic Index, Index of Orthodontic Treatment Need

## Abstract

**Objectives** To investigate the psychosocial impact of malocclusion and self-rated and clinician-rated orthodontic treatment need on young adult patients in the Western Province of Saudi Arabia.

**Materials and Methods** Eighteen- to 30-year-old patients ( *n* =355) attending a tertiary dental care facility were included. Three instruments were used for data collection: (1) Psychosocial Impact of Dental Aesthetic Questionnaire (PIDAQ), (2) aesthetic component of the Index of Orthodontic Treatment Need (IOTN-AC), self-rated and clinician-rated, and (3) clinician-rated Dental Aesthetic Index (DAI). Data analysis included descriptive statistics, Kruskal–Wallis test, Mann–Whitney U-test, and multiple linear regression analysis.

**Results** Females were significantly more impacted than males on all domains with the exception of the dental self-consciousness domain. For both the self-rated and clinician-rated IOTN and the DAI, it was found that the more severe the malocclusion, the higher the impact on all domains except for the dental self-confidence domain, which showed that patients with mild malocclusions were more affected than those with moderate and severe malocclusions. Significant associations were observed between independent variables (age, sex, self-rated IOTN, and DAI) and total PIDAQ score.

**Conclusions** Perceived psychosocial impact of dental aesthetics is directly related to severity of malocclusion (self-rated and clinician-rated) for all domains of the PIDAQ accept the DSC, and females showed higher psychosocial impact than males. Clinicians should consider the impact of malocclusion and certain demographic characteristics on the psychosocial well-being of an individual when determining the orthodontic treatment need.

## Introduction

An individual's personality is significantly shaped by how they perceive their appearance, and when they present with malocclusion, especially anteriorly, negative social responses are triggered which can lead to a poor self-image. 1 2 In fact, malocclusion could even be considered a physical disability as it restricts a person's social relationships, and hence their opportunities. 3 As people inherently feel the need to belong and be accepted by their social network, they are likely to be considerably affected if they do not meet the socially acceptable appearance norms. 4 Even small deviations from the commonly held standards of appearance can influence young adults in particular, leading to reduced self-esteem and a belief that they are inferior to their peers. 1 Such a lack of self-confidence is also a critical issue in that it often affects an individuals' quality of life (QoL) and prevents them from developing an effective professional profile. 5

The field of orthodontics tends to be subject to limited funding, and thus it must often prioritize those patients with the greatest need for treatment. To this end, sociodental indicators offer a way to determine the needs of individual patients. 3 6 In addition, orthodontic patients who do not need immediate treatment can avoid the potential risks posed by unnecessary treatment by assessing their behavior and/or motivation before the start of treatment. 3 6 For example, there is thus far a lack of evidence indicating that patients with minor malocclusions would obtain significant benefit from orthodontic treatment in terms of their dental function and oral health. 7

Nevertheless, patients may request orthodontic treatment out of concern for their appearance. In standard practice, assessing the need for orthodontic treatment is generally based on several psychosocial factors as well as normative assessments through the use of occlusal indices. 6 However, these methods are solely based on the perspective of the dental clinician, bypassing the concerns of the patients themselves. Due to this gap between the perceptions of what constitutes an acceptable dental appearance and whether orthodontic intervention is required, traditional assessment methods have proven to be inadequate. 8 9 10

For example the Dental Aesthetic Index (DAI) and Index of Orthodontic Treatment Need (IOTN), which are considered conventional occlusal indices, can be used to assess a malocclusion in terms of its aesthetic and anatomic characteristics. However, they do not reflect how the malocclusion is causing a degraded self-image that is impacting the patient's QoL regarding their well-being and daily functioning. 11

Previous research has indicated that a patient's psychosocial well-being is strongly related to how they perceive their dental aesthetic appearance. As psychosocial well-being informs the patient's general health, problems with dental aesthetics, as perceived by the patient, are an important factor, particularly as they predict a worsening QoL in terms of oral health. 1 12 As a result, research attention has recently focused on the development of appropriate psychometric tools to specifically measure a patient's oral health-related QoL (OHQoL). One such tool, namely the Psychosocial Impact of Dental Aesthetics Questionnaire (PIDAQ), was developed to measure to what extent the self-image of young adult orthodontic patients is affected by their dental aesthetic appearance. 13 The PIDAQ has been shown to have good reliability and construct validity for adults in international contexts concerning the association between psychosocial dimensions and dental aesthetics (as perceived by the patient). 14 15 16 17 18 However, the literature revealed a limited number of studies that have explored the impact of malocclusion on the psychosocial well-being of the affected individuals and results are conflicted. In addition, how clinician-rated and self-rated malocclusion levels differ in their impact is also unclear and should be further explored.

Thus, the aim of this study was to investigate the psychosocial impact of different grades of malocclusion and self-rated orthodontic treatment need on a sample of young adult dental patients attending a tertiary dental care facility in the Western Province of Saudi Arabia.

## Materials and Methods

This was a cross-sectional study at a public tertiary dental care facility in the city of Al-Madinah Al-Munawwarah, Saudi Arabia. Data collection was conducted over a period of 3 months (May 2021–July 2021) through a convenience sampling method. Ethical approval was granted by Taibah University, College of Dentistry Research and Ethics Committee (TUCDREC/04032021).

### Study Sample

The sample included eligible young adult dental patients 18 to 30 years old who were attending a dental screening appointment for further dental treatment and were able to read and speak Arabic. Patients who studied/studying dentistry and those with a history of orthodontic treatment and/or patients undergoing current orthodontic treatment as well as patients with craniofacial deformity were excluded from the study.

Sample size calculation was performed using Epi Info Software v5.5.6 (U.S. Department of Health and Human Services, Centers for Disease Control and Prevention, United States). The calculation was based on a total population of 660 patients who visited the Dental Hospital for their initial screening appointment during the study period (3 months) and were eligible for inclusion in this study. Expected frequency was set at 17.8% (frequency of moderate and severe malocclusion) and an acceptable margin of error of 5%. A sample of 350 provided power of the study above 99.9%.

### Research Instruments

Patients attending their first visit screening appointment were consecutively recruited and introduced to the study. These screening appointments are usually conducted by dental interns every day of the week during the morning clinical session. If they agree to take part in the study, they were asked to sign a consent form. They were then asked to complete the PIDAQ (Arabic language version), in addition to a brief questionnaire that gathered their demographic details. The PIDAQ instrument comprised 23 items categorized into four sections, namely dental self-confidence (DSC; 6 items), social impact (SI; 8 items), psychological impact (PI; 6 items), and aesthetic concerns (AC; 3 items). The patients self-rated their responses on a 5-point Likert scale (0=not at all, 1=a little, 2=somewhat, 3=strongly, 4=very strongly). Each subscale score can be calculated separately and is obtained by summing the item scores. It is important to note that unlike the rest of the PIDAQ domains, the DSC domain has a positive meaning (i.e., the higher the score the less the impact), and hence, when calculating the total PIDAQ score, the scores of the DSC domain were inverted. 13 A recent study has adapted, psychometrically tested, and validated the PIDAQ's Arabic language version. 16

After completing the questionnaire, the participants were asked to rate their malocclusion level using the IOTN-AC. This instrument involved presenting 10 images depicting malocclusions of the anterior teeth to patients and asking them to rank these according to how well they match their own dental appearance on a scale of grade 1 (the best) to grade 10 (the worst). 19 Subsequently, the patient is categorized into one of three groups according to their IOTN grading: AC grades 1–4 require no orthodontic treatment, AC grades 5–7 have a moderate or borderline treatment need, and AC grades 8–10 have an immediate treatment need. In the current study, the participants were also rated by the clinician using the IOTN-AC grading index.

Finally, the clinician graded the malocclusion level of each participant using the DAI. This index utilized 10 dentofacial anomaly parameters that assess the anterior teeth regarding their clinical and aesthetic features. The DAI consists of 10 occlusal traits related to dentofacial anomalies according to the three components of dentition, spacing–crowding, and occlusion. Scores for each component were multiplied by a previously reported weight and a constant was added to obtain a final DAI score for each participant. 20 Malocclusions were categorized into four grades regarding the recommendations for orthodontic treatment and prioritization: grade 1 is a normal/minor malocclusion with an absent or slight treatment need (DAI ≤ 25); grade 2 is a definite malocclusion with a treatment need that is elective (26 ≤ DAI ≤ 30); grade 3 is a severe malocclusion with a high treatment need (31 ≤ DAI ≤ 35); and grade 4 is a very severe malocclusion with a compulsory treatment need (DAI ≥ 36).

The clinical examinations in this study were performed by the same orthodontist, who received training and calibration in the use of the IOTN-AC and the DAI score dental parameters. The calibration process was conducted before the study to guarantee reliable data collection. Ten patients were examined twice, 1 week apart, by the same experienced orthodontist to calculate the intra-examiner reliability. The results showed high reproducibility with an intraclass correlation coefficient 0.90 for the IOTN-AC and 0.85 for the DAI.

### Statistical Analysis

Descriptive statistics (means, standard deviation [SD], frequencies and percentages) was used to describe the sample's sociodemographic characteristics, malocclusion levels, and the mean PIDAQ scores. To test the impact of each demographic variable, self-rated IOTN, clinician-rated IOTN and DAI on the PIDAQ scores, nonparametric statistics (Kruskal–Wallis and Mann–Whitney) were used as the data did not follow a normal distribution when the normality test was checked with the Shapiro-Wilk test. Multiple linear regression analysis was used to test the influence of the aforementioned variables on the PIDAQ scale and subscales. The significance level was set at *p* <0.05. SPSS 14.0 for Windows (SPSS Inc., Chicago, Illinois, United States) was used for the statistical analysis. To test the internal consistency of the PIDAQ questionnaire, Cronbach's α was calculated for the scale as a whole and for each individual domain. Cronbach's α for the scale was 0.86, thus indicating good internal consistency.

## Results

The total number of participants who completed the questionnaires was 355 out of 400 participants who were invited to participate (88.75% response rate), of which 152 were males and 203 were females. The distribution of the sample's sociodemographic characteristics, including age, sex, marital status, and self-rated and clinician-rated malocclusion levels, is presented in Table 1 .

**Table 1:** Descriptive statistics showing demographics, self-rated malocclusion severity (IOTN), and clinician-rated malocclusion severity (IOTN and DAI) of the sample

Variable	Subcategories	*N* (%)
Age	18–22	165 (45.5%)
	23–26	109 (30.7%)
	27–30	76 (21.4%)
Sex	Male	152 (42.8%)
	Female	203 (57.2%)
Marital status	Single	292 (82.3%)
	Married	45 (12.7%)
	Divorced	9 (2.5%)
	Engaged	9 (2.5%)
Self-rated IOTN	Mild malocclusion	292 (82.3%)
	Moderate malocclusion	44 (12.4%)
	Severe malocclusion	19 (5.4%)
Clinician-rated IOTN	Mild malocclusion	298 (83.9%)
	Moderate malocclusion	39 (11.0%)
	Severe malocclusion	18 (5.1%)
DAI	Minor malocclusion/no treatment needed	298 (83.9%)
	Definite malocclusion/treatment elective	20 (5.6%)
	Severe malocclusion/treatment highly desirable	15 (4.2%)
	Very severe malocclusion/treatment mandatory	22 (6.2%)

Abbreviations: DAI, Dental Aesthetic Index; IOTN, Index of Orthodontic Treatment Need.

Overall, the total mean score (SD) for the PIDAQ in the current study was 37.02 (SD=24.16) with the highest rating given to the dental self-consciousness domain (11.61, SD=7.37) followed by the aesthetic attitude (10.20, SD=9), SI (7.63, SD=6.91), and PI (7.60, SD=5.1) domains, respectively (see Table 2 ).

**Table 2:** Mean and SD for PIDAQ subscale and total scores

PIDAQ domain	Mean	SD	Range
Dental self-consciousness total score:	11.61	7.37	0–24 (24)
Social impact total score:	7.63	6.91	0–24 (24)
Psychological impact total score:	7.60	5.10	0–16 (16)
Aesthetic attitude total score:	10.20	9.00	0–28 (28)
PIDAQ total score	37.02	24.16	0–92 (92)

Abbreviations: PIDAQ, Psychosocial Impact of Dental Aesthetics Questionnaire; SD, standard deviation.

Table 3 displays the influence of different sociodemographic characteristics on the results of the PIDAQ scores. While age and marital status did not show any significant effect on PIDAQ scores, sex on the other hand did, where females were significantly more impacted than males on all domains ( *p* <0.0001).

**Table 3:** Comparison of mean PIDAQ subscale and total scores among different sociodemographic variables

Variable	Subcategories	DSC, mean (SD)	*p* -Value	SI, mean (SD)	*p* -Value	PI, mean (SD)	*p* -Value	AC, mean (SD)	*p* -Value	Total PIDAQ, mean (SD)	*p* -Value
Age	18–22	12.0±7.5	0.134	7.4±7.1	0.724	7.9±5.1	0.652	9.5±8.7	0.437	36.7±15.9	0.302
	23–26	12.1±7.3		7.6±6.5		7.5±5.0		10.4±8.9		37.1±14.9	
	27–30	10.1±7.1		8.2±6.5		7.3±5.3		11.3±9.7		40.1±17.5	
Sex	Male	10.1±7.1	0.001 a	6.0±6.3	0.000 a	6.0±4.5	0.000 a	8.0±8.3	0.000 a	33.2±15.2	0.000 a
	Female	12.7±7.4		8.8±7.1		8.8±5.2		11.8±9.1		40.7±15.8	
Marital status	Single	11.8±7.4	0.411	7.6±6.9	0.942	7.5±5.1	0.118	9.9±8.9	0.550	36.8±15.8	0.341
	Married	10.1±7.1		7.8±7.0		7.2±5.3		11.0±9.9		40.0±17.2	
	Divorced	11.2±8.2		6.9±6.7		8.9±2.7		10.4±8.6		39.0±17.0	
	Engaged	13.6±6.4		8.8±7.1		11.2±4.7		14.0±7.6		44.4±14.2	

Abbreviations: AC, aesthetic concern; DSC, dental self-confidence; PI, psychosocial impact; SI, social impact.

a Statistically significant.

Table 4 displays the influence of different self-rated and clinician-rated malocclusion levels on the results of the PIDAQ score. For both the self-rated and clinician-rated IOTN, it was found that participants with moderate and severe malocclusions were significantly more impacted on all domains including the PIDAQ total score ( *p* <0.0001 and *p* <0.001) except the DSC domain which showed that individuals with mild malocclusion levels were more effected than those with moderate and severe malocclusions ( *p* <0.0001).

**Table 4:** Comparison of mean PIDAQ subscale and total scores among different self-rated and clinician-rated malocclusion severity levels

Variable	Subcategories	DSC, mean (SD)	*p* -Value	SI, mean (SD)	*p* -Value	PI, mean (SD)	*p* -Value	AC, mean (SD)	*p* -Value	Total PIDAQ, mean (SD)	*p* -Value
Self-rated IOTN	Mild	13.3±7.03	0.000 a	6.7±6.4	0.000 a	6.9±5.0	0.000 a	8.6±8.2	0.000 a	35.5±15.4	0.000 a
	Moderate	6.89±5.8		12.0±6.8		10.8±5.0		17.3±7.9		46.9±14.8	
	severe	4.6±5.5		12.3±9.3		10.9±4.3		18.9±9.4		46.7±17.3	
Clinician-rated IOTN	Mild	12.9±7.1	0.000 a	6.9±6.5	0.000 a	7.2±5.1	0.009 a	8.8±8.4	0.000 a	35.9±15.1	0.000 a
	Moderate	8.1±6.9		11.0±6.7		9.7±4.4		17.5±8.5		46.2±17.0	
	Severe	6.8±7.2		12.5±9.2		8.7±5.3		16.9±9.7		44.9±20.3	
DAI	Minor malocclusion	12.7±7.3	0.000 a	7.0±6.5	0.001 a	7.0±5.0	0.000 a	8.9±8.3	0.000 a	35.5±14.8	0.000 a
	Definite malocclusion	10.2±6.5		9.0±7.3		11.0±4.1		14.9±9.0		45.1±16.6	
	Severe malocclusion	10.7±8.0		11.0±8.6		10.6±5.1		16.3±9.2		48.7±20.2	
	Very severe malocclusion	6.8±6.7		13.3±8.0		10.3±4.9		18.9±10.0		49.3±17.6	

Abbreviations: AC, aesthetic concern; DSC, dental self-confidence; PI, psychosocial impact; SI, social impact.

a Statistically significant

This was also the case with the DAI where it was found that those with minor malocclusions showed significantly higher dental self-consciousness scores than those with definite, severe, and very severe malocclusions ( *p* <0.0001). However, for all other domains, it was found that the more severe the malocclusion (i.e., higher treatment need), the greater the impact ( *p* <0.001 and *p* <0.0001).

Table 5 displays the linear regression model analysis which revealed that sex, age, self-rated IOTN, and DAI were significant predictor variables of PIDAQ scores ( *p* <0.05). However, clinician-rated IOTN was not significant at *p* =0.929. The analysis revealed an intermediate *R* -value (0.431) and that the model is capable of explanation of 18.6% of the variance of PIDAQ values. Durbin–Watson and multicollinearity analyses revealed no serial correlation in the model (i.e., Durban–Watson value between 1.5 and 2.5, and tolerance values of more than 0.8 for all variables).

**Table 5:** Multiple linear regression analysis for the association of total PIDAQ score and independent variables

Dependent variable	Independent variables	Unstandardized coefficients		Standardized coefficients	*t*	Sig.	95.0% confidence interval for *B*	
		*B*	Std. error	Beta			Lower bound	Upper bound
Total PIDAQ score a	(Constant)	3.997	6.311		0.633	0.527	−8,414	16.409
	Self-rated IOTN b	4.461	1.611	0.149	2.769	0.006	1.293	7.629
	Clinician-rated IOTN	0.164	1.857	0.005	0.089	0.929	−3.488	3.817
	Age	0.458	0.219	0.102	2.093	0.037	0.028	0.888
	Sex c	6.343	1.585	0.197	4.003	0.000	3.227	9.460
	DAI d	0.430	0.098	0.264	4.389	0.000	0.237	0.622

Abbreviations: DAI, Dental Aesthetic Index; IOTN, Index of Orthodontic Treatment Need.

Note: Dependent variable: PIDAQ scores.

a As PIDAQ increases, psychosocial impact of malocclusion increases.

b As Self -rated IOTN increases, need for treatment increases.

c Sex: 0=boy, 1=girl.

d As DAI increases, need for treatment increases.

## Discussion

Over the past four decades there has been increasing interest in patient-centered outcome studies especially when evaluating OHQoL. This has been stressed by the World Health Organization as an area of interest and is considered as one of the factors that resulted in an increase in QoL research across many fields including the dental field. As more and more OHQoL studies are conducted, it has become apparent that factors such as oral health, malocclusion, and treatment can significantly affect a patients' QoL. 21 Although the impact of malocclusion on the OHQoL is well documented, there is still conflicting evidence about the impact of malocclusion on the psychosocial well-being of an individual and how clinician-rated and self-rated malocclusion levels differ in their impact. The current study evaluated the impact of self-rated and clinician-rated malocclusion levels on the psychosocial well-being of a young adult population using the Arabic version of the PIDAQ instrument which has been previously cross-culturally adapted and validated for an Arabic-speaking population. 16

The mean PIDAQ score obtained for the study sample was 37.8±15.76, which, when compared with previous studies including those with a similar population sample, is considered low indicating a low impact of malocclusion on the psychosocial well-being of the participants. 14 22 23 This could be due to the fact that the majority of the sample had a mild malocclusion (as rated by the IOTN) or no treatment need (as rated by the DAI), which may have resulted in lower psychosocial impacts.

The results did not show any significant association between PIDAQ scores and marital status, which is contradictory to previous studies that found that those who are married/in a relationship showed higher DSC scores, whereas those who were single were significantly impacted in the SI and PI domains. 15 The reason for the contradictory results may be due to the small number of participants who were married compared with those who were single in the current study sample, which one would expect considering that the majority of the participants were between the ages of 18 and 26.

Similarly, age did not show significant association with the PIDAQ scores, which agrees with several studies. 16 18 The age intervals used for the current study were not completely equal, where the first group was 18 to 22 (5 years) while the other two groups were 22 to 26 (4 years) and 27 to 30 (4 years), which may be a contributing factor for the lack of association. However, this interval was used following previous studies to enhance comparability. 16

With regard to gender, the results revealed that females were significantly more impacted on all domains than males. This is not a rare finding as several studies have reported that malocclusion might have significantly more psychosocial impact on females compared with males. Studies that have examined the association between aesthetics and gender have concluded that women are generally more demanding with regard to beauty and aesthetics, more affected by what they consider facial and corporal aesthetic defects, and more critical in general regarding everything related to aesthetics. 1 5 18 24 25

As for the effect of malocclusion on the psychosocial well-being of the sample, our study confirms that the more severe the malocclusion is, the higher the impact on all domains including the PIDAQ total score, with the exception of the DSC domain which showed a negative correlation. While previous studies have reported that higher orthodontic treatment need resulted in higher negative psychosocial impacts and a worse QoL compared with those who have no treatment need, it is unclear why this does not apply to the DSC domain in the current study. 22 26 One explanation could be that those with low need for treatment have malocclusions that are not severe enough to cause impacts on the psychological, social, and aesthetic domains however, resulted in them being more aware and specific about the appearance of their teeth.

Multiple linear regression analysis showed that sex, age, self-rated IOTN, and DAI were predictors associated with psychosocial impacts confirming the results of previous studies that psychosocial impacts of dental aesthetics are multifactorial and are influenced by measures of normative orthodontic treatment need as well as subjective aspects. 18 27 The regression models' global fit was summarized using the *R* 2 values, which reflect to what extent the proportion of the PIDAQ's scale variability can be attributed to a linear combination of the explanatory variables selected in this study. The results showed that the explanatory variables explained around 18.6% of the overall PIDAQ scale variation. However, the interpretation of the results based on the *R* 2 values demands some degree of caution as a significant portion of the variability can be due to unidentified variables or inherent variability in the data. In other words, caution must be used in assessing how significant the independent variables are in predicting the self-perceived psychosocial impact of dental aesthetics for young adults, and the individual patient's psychological and clinical aspects must also be taken into account.

On interpreting the results of the present study, it is important to highlight its limitations. Although the PIDAQ scale was psychometrically tested and cross-culturally adapted for a Saudi population, little is known about the stability of the scale over time and could be a study objective of future projects. In addition, the study was single-centered and hence, generalizability of the results should be considered with caution. However, the sample size was considered sufficient to overcome the selection bias. It is also important to note that the majority of the participants had malocclusions that were considered mild or no need for treatment, this could be because both the AC of IOTN and the DAI may not adequately capture all malocclusion traits and hence it may prove beneficial to include more detailed means of measuring malocclusion. Nevertheless, perceived dental and facial aesthetics is multifactorial and might be affected by other variables that were not examined in the study whether in the past or how aesthetically demanding their current social status is (e.g., lifestyle, careers, etc.), which could be an interesting area for future research.

## Conclusion

Perceived psychosocial impact of dental aesthetics is directly related to severity of malocclusion (self-rated and clinician-rated) for all domains of the PIDAQ with the exception of the DSC domain. Age, sex, self-rated IOTN, and clinician-rated DAI are significant predictors of the total PIDAQ score. Clinicians should consider the impact of malocclusion and certain demographic characteristics on the psychosocial well-being of an individual when determining the orthodontic treatment need.
